# Appendicitis

**Published:** 1902-07-05

**Authors:** 


### Hospital Clinics and Medical Progress.

### APPENDICITIS.

Events of national importance have conspired to give peculiar interest to the Cavendish Lecture delivered so recently as June 20th by Sir Frederick Treves before the West London Medico-Chirurgical Society, for on that occasion he took for his theme inflammation of the vermiform appendix, a malady for the treatment of which he was within a week called upon to perform an operation upon his Majesty the King. A point of primary importance in regard to the whole question is, what do we mean by an attack of appendicitis? for one can hardly doubt that much of the difference between the views held by various people in regard to treatment depends upon differences of opinion as to the pathological conditions which may be properly included under the title appendicitis. About cases in which the appendix is obviously inflamed, matted, tied down, perhaps surrounded with pus, of course there is no doubt. But how far one can go in the other direction, and how far one may include in the list cases in which the appendix merely contains accumulations of epithelium or micro-organisms, is another matter. Many a time, for example, an appendix which has been removed has shown no external signs of disease. Yet on dissection it has been found to be stuffed with material crowded with micro-organisms, and to these the surgeon has pointed in triumph as proof that if the operation had not been performed a catastrophe would surely sooner or later have arrived; and there is no doubt that the proof which has recently been offered that these so-called catarrhal appendices are in fact highly charged with septic material has in the minds of many given much support to the modern teaching that all appendices which have given rise to symptoms ought to be removed. Sir Frederick Treves, however, gives a very different definition of appendicitis. According to him, the first point to be insisted upon is that the disease with which we are now familiar under the name of "an attack of appendicitis" is a pure peritonitis. There is no consideration about it which is apart from peritonitis. Until the peritoneum is involved it does not exist. We must throw aside all terms as to twisting or turning of the appendix. Quite extensive changes may take place in the appendix without the production of a single symptom: it may be almost obliterated without the production of a single symptom of appendicitis, its mucous membrane can be entirely destroyed, and it can become stenosed or shrunken without the production of a symptom; in fact the grossest changes may be accidentally discovered in it in the course of operations done on other organs, as for example in ovariotomies in patients who have never had any symptoms of appendicitis. "An acute attack of appendicitis is an attack of peritonitis." The word appendicitis merely indicates the point of origin of the peritonitis.

The symptoms connected with the appendix which may precede an attack of appendicitis Sir Frederick describes under three heads. First, he says that extensive changes may, as just mentioned, exist without producing symptoms of any kind. Secondly, that the patient may have minor seizures of pain in the cæcal region, of short duration and of irregular appearance, which have been described under the quite unsuitable title of "appendicular colic." In a few of these cases it is possible that there is a minute infection of the serous membrane and an infinitesimal peritonitis. Lastly, the patient may have an abiding trouble in the right iliac fossa, which may or may not be associated with acknowledged attacks of appendicitis. But in these and other cases of uncomplicated inflammation of the appendix any advance of symptoms from mere discomfort to acute pain implies an advance of the mischief from the inner coats to the peritoneal surface. It is rather a splitting of words, perhaps, but it seems evident that Sir Frederick Treves draws a distinction between an inflammation of the appendix and an "attack of appendicitis."

It has often been said that appendicitis is a new disease, and if we consider the extraordinary frequency with which it is now diagnosed, and remember that twenty years ago the affection was unknown, we need not wonder at this idea obtaining currency. Nevertheless, as Sir Frederick Treves says, "under no circumstances is appendicitis to be regarded as a new disease. It is probable that even the cave-man, with his rudimentary methods of eating, suffered occasionally from appendicitis. The disease is not new but newly discovered." Nevertheless, there is much in what Sir Frederick says concerning the causes which conduce to appendicitis to explain what is probably the fact, namely, the greater prevalence of the disease in modern times; for one of the distinguishing features of modern man is the poorness of his teeth, and one of the commonest causes of appendicitis seems to be the habit of bolting half-chewed food. "If there is one solitary factor in the production of appendicitis which is overwhelming," says Sir Frederick, "it is a loaded cæcum. I really think it is a little exaggeration, but not a gross one to say, that if loading or overloading of the cæcum could be avoided there would be exceedingly little appendicitis. This is so almost uniform a feature of the trouble that one need hardly go into the history of some of the cases. You know what these histories are—a child with teeth overlapping, a man with no masticating teeth to eat meat, the commercial traveller who has his meals all over the country and eats and drinks and smokes too much, and a man who habitually bolts his food." Nothing plays so important a part in the prophylaxis of appendicitis as keeping the cæcum free from indigestible and undigested food.

In regard to treatment Sir Frederick says that there are certain people who assert that you must operate in every case of appendicitis as soon as the diagnosis is made. Others only operate on compulsion. They say "No, the majority of the patients get well," and they only operate in exceedingly acute cases in which pus is evident, or in cases that are really spun out to such a length that they feel there must be some condition within the iliac fossa which can only be reached by operation. Those are the two sides of the question In discriminating between these extreme views Sir Frederick points out first that the analogy which some have sought to draw between perforation or gangrene of the appendix and the same process as affecting the stomach or the ileum is absolutely unjustifiable. "It is monstrous. Not only can a perforation of a fair size occur in the appendix, but a fair degree of gangrene may be met with without causing serious symptoms." Secondly, he insists on the fact that the very great majority of all cases of appendicitis get well spontaneously. Taking the whole series of cases which come under the care of a man in general practice the death-rate may be taken at 5 per cent. Thirdly, he says that operation during an acute attack of appendicitis is attended with great risk to life. The exact figures are not easy to get hold of, but he says that he has been at infinite trouble to go through the records by hundreds, and the death-rate in cases of operation during an acute attack comes out at about 20 per cent. Lastly, there is the very important fact that the removal of the appendix during the quiescent period is attended with infinitely little risk, a fact which he emphasises by saying that since the year 1887 he has removed the appendix during the quiescent period over one thousand times, and that out of that number he has lost but two patients. With these facts before us, Sir Frederick Treves holds that the dictum that operation should be done as soon as the diagnosis is made cannot be supported. On the other hand, in all the ultra-acute cases an immediate operation should be performed. Of these cases there are two perfectly distinct types. In one type the local manifestations are not very striking, the overwhelming symptoms being those of the introduction into the system of a gigantic dose of poison—a temperature of 104° or 105° Fahr., while the pain is not very striking. These are cases of acute septicæmia, the patient dying within 36 or 48 hours, and although in such cases the operation will probably do no good it can do no possible harm, and it should as a matter of positive routine be carried out. In the other class of acute cases the condition is strictly parallel to perforation of the stomach, and no one can have any difficulty in recognising the acute peritonism. These ultra-acute cases cannot be operated on too soon. Finally, an immediate operation should be carried out as soon as there is any suspicion of pus. One does not require a demonstration of pus, but a reasonable suspicion of it. If then one excludes all the ultra-acute cases and those in which there is pus, what about the remainder? As to these, Sir Frederick says "it is seldom imperative that an operation should be discussed until about the fifth day. . . . If the temperature comes down and is getting towards the normal about the fifth day your anxiety is becoming comparatively slight," but if the temperature keeps up after that day the local condition will probably have to be dealt with. He therefore makes the "somewhat moderate suggestion that the surgeon should hold his hand until such time as the fifth day has been reached when the requirements of the case will have been made quite manifest."

Leaving now the question of acute appendicitis, one comes to that of the treatment to be advised in regard to a patient who has had an attack from which he has recovered without operation. In view of the trifling danger attending an operation in the interval, one might say that in such a case one ought to advise removal of the appendix. But that does not entirely cover the whole problem. The question hinges much on the probability of relapse, and Sir Frederick says that, having now during a considerable number of years seen some thousands of cases of appendicitis, he is able to state that he has seen a great many patients who have had only one attack. Hence the habits of the patients become a matter of some importance, and if it should be found that the attack has come on in a man who has many gross errors of diet to correct, it may be desirable to see whether by dint of correcting these bad habits recurrence may be prevented. Chronic appendicitis, however, in all cases demands removal of the appendix.

